# Mitochondrial function in macrophages controls cardiac repair after myocardial infarction

**DOI:** 10.1172/JCI167079

**Published:** 2023-02-15

**Authors:** David Weissman, Christoph Maack

**Affiliations:** 1 Comprehensive Heart Failure Center (CHFC), University Clinic Würzburg, Würzburg, Germany.; 2 Medical Clinic 1, University Clinic Würzburg, Würzburg, Germany.

## Abstract

Cardiac healing following acute myocardial infarction (MI) involves the mobilization and activation of immune cells, including macrophages. In the early phase after MI, macrophages adopt a proinflammatory phenotype, while polarizing toward a reparative one in the late stage. Although metabolic reprogramming has been observed during this transition, the mechanistic links to macrophage differentiation are still poorly understood. In this issue of the *JCI*, Cai, Zhao and colleagues demonstrate that mitochondrial function in macrophages governed the resolution of inflammation and tissue repair by modulating the phagocytic removal of apoptotic cells (so-called efferocytosis) as well as myofibroblast activation. These findings provide important mechanistic insights into the potential relevance of metabolic modulation of macrophage functions following MI, which might lead to alternative therapeutic strategies for MI.

## The role of immune cells in cardiac repair after MI

**Figure 1:**
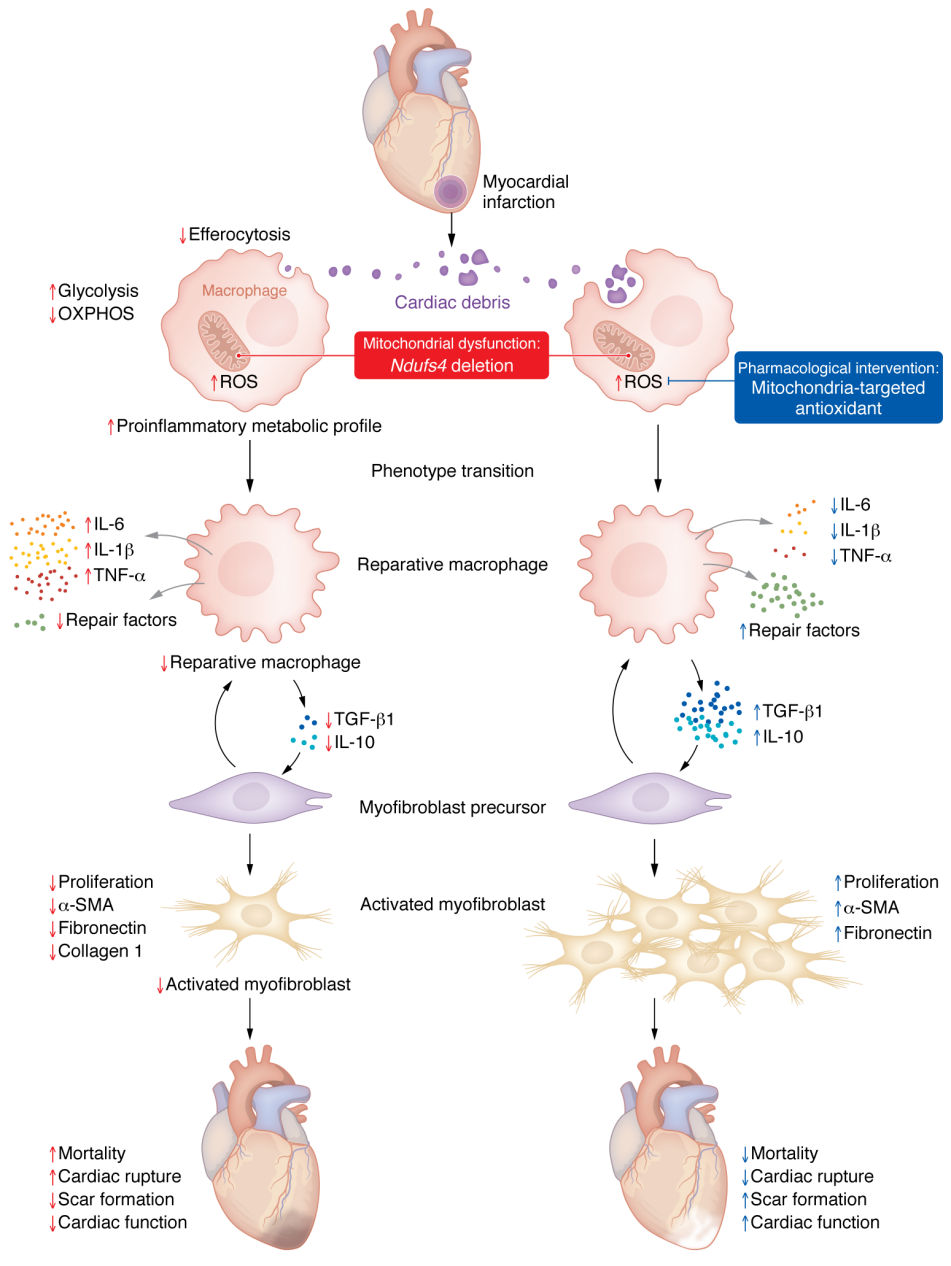
Myeloid-specific deletion of the mitochondrial complex I protein *Ndufs4* compromises macrophage function during cardiac repair after MI. Following acute MI, immune cells, including monocyte-derived macrophages, remove cell debris through efferocytosis but can also amplify the inflammatory response by secreting proinflammatory mediators. Subsequently, proinflammatory macrophages transition from a proinflammatory to a reparative phenotype, a process that associates with metabolic remodeling: shifting from relying on glycolysis (associated with the proinflammatory phenotype) toward mitochondrial OXPHOS (associated with the reparative phenotype). This transition is characterized by increased secretion of antiinflammatory cytokines, chemokines, and repair factors that stimulate the activation and proliferation of cardiac myofibroblasts, which promote tissue repair and scar formation. In this issue of the *JCI*, Cai, Zhao, and co-authors (20) report that mitochondrial dysfunction in macrophages, induced by *Ndufs4* deletion, can cause excessive mitochondrial ROS production, which primes the cells to a greater inflammatory response while impairing efferocytosis. This process, in turn, blunts macrophage polarization, reducing the reparative phenotype, and suppresses the proliferation and activation of myofibroblasts, resulting in poor wound healing and increased mortality. Interestingly, mitochondria-targeted ROS scavenging improved efferocytosis and myofibroblast functions, resulting in reduced infarct rupture and mortality.

Cardiac repair following myocardial infarction (MI) is the result of a finely orchestrated series of events, which includes an initial inflammatory phase, characterized by intense sterile inflammation and subsequent mobilization of innate and adaptive immune cells, in particular neutrophils, monocytes, and macrophages, to the infarcted area (1). This response is followed by the inflammation resolution phase, marked by myofibroblast activation, tissue healing, and scar formation (Figure 1). Neutrophils are the first cells to populate the infarcted area, followed by proinflammatory monocytes and macrophages, which are both derived from circulating monocytes (1, 2). Together, neutrophils, monocytes, and macrophages degrade extracellular matrix (ECM) constituents and clear apoptotic cells by phagocytosis, a process termed “efferocytosis” (1, 3) (Figure 1).

During the resolution phase, macrophages transition from the proinflammatory to a reparative phenotype (4), promoting the activation of cardiac myofibroblasts (5) and endothelial cells, which are both critical for ECM remodeling and post-MI reparative angiogenesis (Figure 1) (6, 7). Therefore, a better understanding of the molecular mechanisms orchestrating such macrophage phenotype transition may pave the way for interventions that improve post-MI cardiac repair and function and resolve other inflammatory conditions.

## Macrophage immunometabolism in cardiac injury and repair

Emerging evidence suggests that metabolic remodeling is central to macrophage polarization and its effector functions (8). In proinflammatory macrophages, glycolysis is upregulated, while mitochondrial oxidative phosphorylation (OXPHOS) is suppressed. This phenotype associates with functional mitochondrial remodeling, including a repurposing from ATP synthesis to ROS production. In this context, the mitochondrial Krebs cycle is disrupted at several steps, rewiring metabolic flux (9). Mechanistically, glycolysis represents an oxygen-efficient pathway for rapid ATP production in proinflammatory macrophages, especially in hypoxic conditions that characterize the infarcted myocardium. However, glycolysis also provides the necessary metabolites required to support the augmented biosynthetic demand and functional changes associated with macrophage effector functions (10).

In contrast, reparative macrophages produce ATP predominantly via OXPHOS, fueled by an intact Krebs cycle, while reducing their glycolytic activity (9). Interestingly, metabolites derived from efferocytic cargo can fuel mitochondrial respiration and thereby govern the NADH/NAD^+^ redox state, in turn driving macrophage reparative polarization. These findings are indicative of a role for efferocytosis-derived metabolic signaling in inflammation resolution (11).

Despite prior in vitro evidence that functional macrophage phenotypes are linked to distinct metabolic profiles, more recent studies have progressively resolved the mechanistic links between macrophage metabolism and phenotype transition during MI. Using time-dependent differential transcriptomic gene expression in macrophages isolated from infarcted mouse hearts, Mouton et al. (10) previously revealed that in the early post-MI phase (day 0 and day 1), genes encoding glycolytic enzymes are upregulated in macrophages, in concert with genes encoding ECM-degrading enzymes and proinflammatory mediators. In contrast, during the late phase after MI (days 3 to 7), macrophages upregulate genes that promote mitochondrial OXPHOS, including succinate dehydrogenase, mitochondrial electron transport chain (ETC) assembly factors, and cristae formation. Furthermore, genes involved in ECM remodeling and scar formation are also activated (10). In a rat model of MI, inhibition of macrophage glycolysis ameliorates inflammation and improves cardiac function (12). Together, these observations support the notion that metabolic and mitochondrial functions of macrophages play a pivotal role in the transition from a proinflammatory toward a reparative phenotype after MI.

## Mitochondrial function and ROS production in cardiac injury

Mitochondrial integrity and functions are central for macrophage effector functions. On the one hand, mitochondria are important for ATP production (via mitochondrial OXPHOS), but they are also involved in the initiation of different signaling pathways, including those for cell growth, proliferation, and differentiation (13). Furthermore, mitochondria are a major source of ROS in macrophages. Mitochondrial ROS (mROS) are considered a normal byproduct of the activity of the mitochondrial ETC, however mROS also mediate redox-dependent activation of molecules involved in both physiological and pathological conditions (13).

The mitochondrial ETC consists of four interconnected complexes, including complex I (or NADH: ubiquinone oxidoreductase), complex II (or succinate dehydrogenase), complex III (or cytochrome bc1 complex), and complex IV (or cytochrome c oxidase), as well as the electron carriers ubiquinone and cytochrome c. Together, they couple electron flux along the ETC to translocate protons across the inner mitochondrial membrane. This electrochemical gradient drives ATP synthesis at complex V (F_1_F_0_ ATP synthase) (14). Complexes I and III are the major sources of ROS that contribute to the activation of different proinflammatory signaling pathways (8, 15).

In fact, excessive mROS activate the NF-κB signaling pathway, promoting proinflammatory macrophage polarization (15, 16). Furthermore, mROS can also sustain redox-dependent activation of the MAPK pathway, resulting in uncontrolled cell proliferation and chronic proinflammatory gene expression (17). Finally, abnormal mROS production can activate the nod-like receptor (NLR) family pyrin domain–containing 3 (NLRP3) inflammasome, promoting inflammatory cell death (via pyroptosis), which aggravates cardiac damage and impairs cardiac repair after MI (18, 19). Interestingly, scavenging mitochondrial ROS blunts LPS-mediated proinflammatory macrophage polarization, while promoting antiinflammatory cytokine production (9). These findings suggest that mROS influence the macrophage phenotype, hence modulating the resolution of inflammation and tissue healing.

## Insights into the role of mitochondria in cardiac repair

In this issue of the *JCI*, Cai, Zhao, and colleagues (20) provide evidence of a role for mitochondria in inflammation resolution and tissue repair by modulation of macrophage efferocytosis and crosstalk with fibroblasts. In a careful experimental approach, the authors observed that bone marrow–derived macrophages (BMDMs) with genetic deletion of mitochondrial complex I protein *Ndufs4* (mKO) had reduced mitochondrial function and an increased glycolytic rate, recapitulating the metabolic profile of proinflammatory macrophages (Figure 1). Furthermore, the mKO defect in complex I of the ETC provoked elevated mROS production in macrophages. Although proinflammatory markers were unaltered at baseline, cytokine production was potentiated upon LPS treatment. These data suggested a link between mitochondrial dysfunction and modulation of the inflammatory response.

To address the in vivo importance of their findings, the authors subjected mKO mice to MI and assessed cardiac repair and function (20). After MI, mKO mice showed more pronounced left ventricular (LV) dysfunction, thinner myocardial scars, and an increased infarct size compared with controls. These results suggest that mitochondrial dysfunction in macrophages promoted cardiac injury and impaired scar formation. Subsequently, the authors investigated the antiinflammatory profile of mKO mice after MI. One day after the MI, mKO mice had higher levels of neutrophils and proinflammatory chemokines and cytokines in their plasma and the infarcted area, whereas no differences in monocyte and macrophage content were observed. At day 7, fewer monocytes and macrophages were observed in the infarcted area of mKO mice, but the levels of proinflammatory mediators and apoptotic markers were increased. These data suggest that mitochondrial defects impair and/or delay the transition of infiltrating macrophages to a reparative phenotype during the healing phase (Figure 1).

To gain further mechanistic insights, Cai, Zhao, and co-authors (20) elegantly examined macrophage efferocytic function while characterizing phenotypic polarization. Compared with controls, mKO macrophages had impaired efferocytosis and a lower percentage of CD206^+^ cells, which indicated a reparative phenotype and resulted in reduced CD206^+^ cell accumulation in the infarcted area after MI. Together, these data support the notion that efferocytosis contributes to reparative polarization, fostering tissue repair and the resolution of inflammation (Figure 1). In addition, mitochondrial complex I deficiency in macrophages impaired the proliferation and activation of cardiac myofibroblasts following MI, which was likely related to impaired regulatory crosstalk between macrophages and cardiac fibroblasts (Figure 1).

Finally, applying a mitochondria-targeted antioxidant to mKO mice in vivo restored efferocytosis, gene expression of antiinflammatory cytokines, and myofibroblast activation, thereby preventing cardiac rupture (20). These data highlight that excessive mitochondrial ROS, resulting from mitochondrial dysfunction in macrophages, compromise myocardial repair after MI.

## Concluding remarks and future directions

Cai, Zhao, and co-authors (20) need to be congratulated for addressing an important scientific question with substantial clinical implications. The role of metabolism and mitochondrial function in inflammation is receiving increased interest, and this study elegantly provides mechanistic insights into the role of mitochondrial function in macrophage transition from a proinflammatory to a reparative phenotype. This transition occurs through modulation of efferocytosis and crosstalk between mitochondria and fibroblasts, which is critical for the optimal post-MI healing response, as well as for other inflammatory conditions.

Despite these insights, further investigation is required to determine whether increased mROS in macrophages have a role in the development of heart failure independent of wound healing. In addition, the possibility that mitochondria-targeted ROS scavenging in other cell types may have also contributed to the observed effects warrants further study. Finally, considering that (a) the gene deletion technique used in the work of Cai, Zhao, and colleagues affects both neutrophils and monocytes/macrophages; (b) the data showed an increased amount of neutrophils in the early post-MI phase in mKO mice; and (c) CXCL1/2 levels were increased in the infarcted area of mKO hearts (which can mobilize inflammatory monocytes), it is possible that mitochondrial dysfunction in other immune cells (e.g., neutrophils) also affected the functions of those cells and contributed to the poorer post-MI outcomes observed in the mKO mice. Therefore, similar studies focused on the role of mitochondrial dysfunction in other immune cells, such as neutrophils, will expand our understanding of the mechanisms described in the current work (20).

Taken together, the findings of Cai, Zhao, and co-authors (20) illustrate the importance of dynamic changes in immune cell metabolism that modulate their effector functions and suggest that mitochondrial function may be an attractive therapeutic target in the setting of cardiac injury and/or other inflammatory conditions. Future studies that focus on clarifying these intriguing interfaces will highlight a role for multidisciplinary research that extends beyond fundamental topics and includes translational issues.

## Version 1 02/15/2023

Electronic publication
